# Mitochondrial complex I deficiency in a 4-year-old boy due to compound heterozygous NDUFV1 mutation: a case report of a new pathogenic variant

**DOI:** 10.1093/omcr/omae166

**Published:** 2025-04-08

**Authors:** Salim Haddad, Elie Salloum, Abdullah Silan, Gazel kalecioğlu, Maria Abdulnour, Sultaneh Haddad, Diana Alasmar, Mahmoud Alayash, Ahmed Noman Ghaleb

**Affiliations:** Faculty of Medicine, Damascus University, Damascus, Syrian Arab Republic; Faculty of Medicine, University of Heidelberg, Heidelberg, Germany; Faculty of Medicine, Tishreen University, Latakia, Syrian Arab Republic; Faculty of Medicine, Aleppo University, Aleppo, Syrian Arab Republic; Faculty of Medicine, Damascus University, Damascus, Syrian Arab Republic; Children’s University Hospital, Damascus, Syrian Arab Republic; PhD of inherited metabolic diseases at Damascus University; Faculty of Medicine, Damascus University, Damascus, Syrian Arab Republic; Children’s University Hospital, Damascus, Syrian Arab Republic; Sulaiman Alrajhi University, Saudi Arabia

**Keywords:** mitochondrial complex I deficiency, mitochondrial diseases, NDUFV1 mutation, white matter

## Abstract

Mutations in the NDUFV1 gene are associated with mitochondrial complex I deficiency and have been linked to various clinical conditions such as Leigh syndrome, severe infantile lactic acidosis, newborn cardiomyopathy, progressive leukoencephalopathy, and other encephalomyopathies. Genetic alterations revealed mitochondrial complex 1 deficiency, nuclear type 4 |AR: two compound heterozygous missense mutations in the NDUFV1 gene, c.640G < A (p.E214K) chr11:67377981 (Exon 1) and c.248C < T (p.S83L) chr11:67376115 (Exon 3) gene. Our case identifies a previously unknown pathogenic effect of the variant ‘c.248C > T’ in the NDUFV1 gene, observed in a 4-year-old boy with left-sided facial paralysis and balance impairment. While this discovery is significant, further exploration of NDUFV1 gene variants is essential for a comprehensive understanding and effective treatment strategies.

## Introduction

The respiratory chain of the mitochondria consists of four electron transport protein complexes (complex I–IV) that are membrane-bound and complex V, an ATP synthase that gives cells energy by phosphate oxidation [1]. With 45 subunits encoded by both nuclear and mitochondrial DNA, Complex I is the biggest complex in the oxidative phosphorylation cycle [2]. Complex I (NADH ubiquinone dehydrogenase) (CI) dysfunctions are genetically and clinically heterogeneous, accounting for a third of all early-onset mitochondrial diseases [3]. The central catalytic core of Complex I (CI) consists of three main modules: N, P, and Q. The P module transports protons across the membrane, the Q module (output module) decreases ubiquinone, and the N module (input module) oxidizes NADH. The NADH dehydrogenase (ubiquinone) flavoprotein 1 (NDUFV1) subunit, which is a part of the N module attaches itself to the flavin mononucleotide, a structure that facilitates the passage of electrons from NADH to iron–sulfur clusters [4]. Complex I deficiency has been linked to pathogenic mutations in the NADH dehydrogenase (ubiquinone) flavoprotein 1 ‘NDUFV1’ gene, which codes for a 51-kD subunit of complex I [2]. The NDUFV1 gene is located on chromosome 11, and there were five clinical categories linked to mitochondrial complex I deficiency (MCID): Leigh syndrome, severe infantile lactic acidosis, newborn cardiomyopathy, progressive leukoencephalopathy, and an unspecified group of other encephalomyopathies [3]. To date, sixteen pathogenic mutations in NDUFV1 have been identified such as c.640G > A (p.Glu214Lys), c.1268C > T (p.Thr423Met), c.766C > T (p.Arg256Cys), c.1118 T > C (p.(Phe373Ser) and c.1156C > T (p.(Arg386Cys) [3–5]

In this report, we presented a 4-year-old child with a compound heterozygous mutation in the NDUFV1 Gen (chromosome 11) consisting of the variants:c.640G > A (p.E214K) in Exon 1 and c.248C > T (p.S83L)in Exon 3. The latter variant has not been yet identified as pathogenic.

## Case presentation

**Figure 1:**
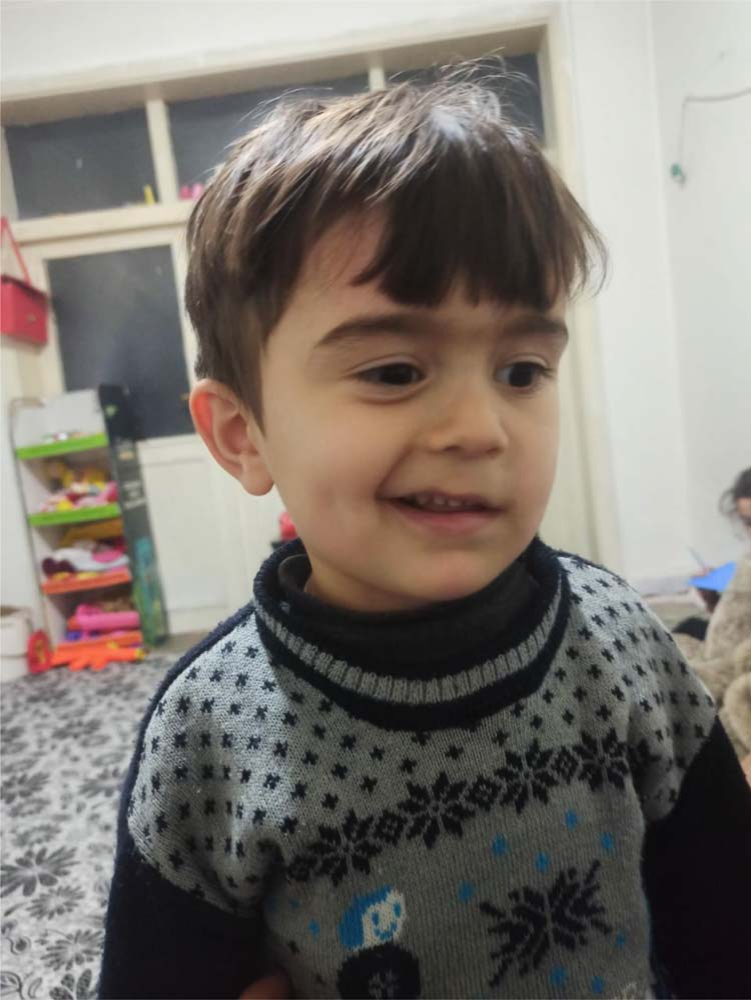
Facial paralysis on the left side.

**Figure 2:**
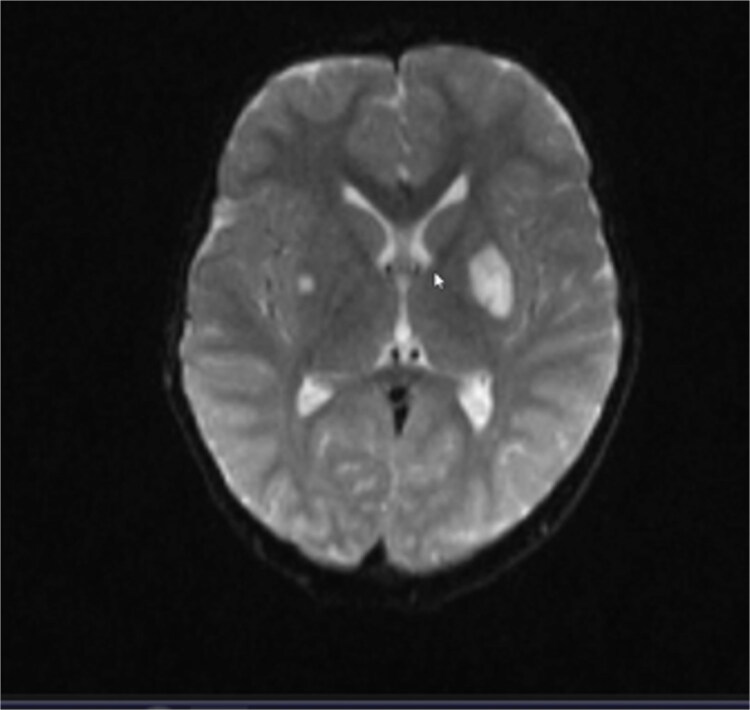
Brain MRI unveiled the presence of diffuse periventricular white matter lesions.

A 4-year-old male child of non-consanguineous parents with a medical history of recurrent hospitalizations since the age of two years, primarily attributed to inhalation incidents and repetitive respiratory infections, presented with a chief complaint of progressive left-sided facial paralysis, characterized by a central pattern. Clinical examination confirmed the manifestation of facial paralysis on the left side (Fig. 1). Laboratory investigations indicated findings suggestive of mitochondrial disease, as evidenced by elevated levels of lactate and alanine in both serum and cerebrospinal fluid. Subsequent brain MRI unveiled the presence of diffuse periventricular white matter lesions (Fig. 2).

Approximately two months following the initial presentation, the child exhibited a progression of symptoms, including increasing weakness in the right forearm and leg, coupled with a notable impairment in balance.

The genetic variations associated with the maturity-onset diabetes of the young (MODY) and their correlation with OMIM and HPO genes were investigated. The analysis included exon-intron junctions (±10 bp), and the obtained data were classified based on pathogenicity according to the ACMG Guidelines (PMID:25741868). Secondary findings (incidental) were also analyzed following ACMG SFv3.1 (PMID:35802134).

## Result

Genetic alterations potentially associated with the referral indication are presented in Table 1, which revealed mitochondrial complex 1 deficiency, nuclear type 4 |AR: two compound heterozygous missense mutations in the NDUFV1 gene, c.640G < A (p.E214K) chr11:67377981 (Exon 1) and c.248C < T (p.S83L) chr11:67376115 (Exon 3) gene.

**Table 1:** Whole exome sequencing (WES) analysis of the child genome.

Transcript	Position	Gene-related disease	Variant	Genotype
NDUFV1 NM_007103.4	Chr11:67377981(Exon 1)	Mitochondrial complex 1 deficiency, nuclear type 4 lAR	Missense Variant, Nmd Transcript Variant c.640G > A (p.E214K) rs121913661	Heterozigot
	Chr11:67376115 (Exon 3)		Missense Variant c.248C > T(p.S83L) rs779150755	Heterozigot

Regarding the diagnosed condition, the coding regions of the RANBP2 gene have a target coverage rate of over 95% with a read depth of 20X, and no pathogenic changes related to this gene have been detected.

Whole Exome Sequencing (WES) analysis of the parental genomes revealed the presence of a nucleotide substitution, specifically c.640G > A (resulting in the p.E214K amino acid change), segregating from the paternal lineage. Additionally, another nucleotide substitution, c.248C > T (leading to the p.S83L amino acid alteration), was identified as originating from the maternal lineage.

Unfortunately, there is no cure for Mitochondrial complex 1 deficiency. Therefore, a group of vitamins and treatments such as riboflavin, biotin, thiamine, levodopa, levocarnitine, and coenzyme Q10 were given to reduce the speed of the disease’s progression.

There is no progression in the child’s situation until the date of writing.

## Discussion and literature review

The most common cause of hereditary mitochondrial illness in children is mitochondrial complex I deficiency, which accounts for up to 30% of cases [6, 7]. Complex I is made up of seven mitochondrial genome-encoded subunits and 37 nuclear genome-encoded subunits [7]. Disease-causing mutations have been discovered in 11 nuclear-encoded complex I genes [MIM: 252010], including NDUFV1 [5]. Mutations in various components of complex I cause pathology in multiple ways, including slowing NADH metabolism and increasing the formation of reactive oxygen species. Moreover, it interferes with the function or assembly of other mitochondrial respiratory chain components [8]. Complex I deficiency is clinically variable, although the majority of affected individuals acquire symptoms during their first year of life and have a rapidly progressing disease history that leads to death in childhood. Clinical manifestations, however, can range from fatal neonatal lactic acidosis to infantile-onset Leigh syndrome, childhood-onset mitochondrial encephalomyopathy, lactic acidosis, and stroke-like episodes (MELAS) syndrome, and in some cases, adult-onset encephalomyopathic syndromes of varying severity [6]. However, these elevations might only occur in specific situations like metabolic crises or periods of stress. Additionally, mitochondrial disorders have been linked to high levels of proline, glycine, and sarcosine [9]. Measuring blood levels of total and free carnitine, coupled with acyl-carnitine profiling, allows the detection of defects in fatty acid oxidation, certain primary aminoacidurias, and organic acidemias. Furthermore, this analysis helps identify secondary fatty acid oxidation defects and carnitine deficiency, which may manifest in primary OXPHOS disorders. MRS can effectively complement the assessment of suspected mitochondrial disease by detecting various compounds related to mitochondrial physiology through variations in their chemical properties within electrical fields. Laboratory techniques for identifying known point mutations encompass PCR coupled with RFLP analysis for singular mutation screening and multiplex PCR with ASO analysis for concurrent detection of multiple known mutations [10]. In our case, the WES test for the patient revealed two compound heterozygous missense mutations, c.640G < A (p.E214K) and c.248C < T (p.S83L) in the NDUFV1 gene. WES analysis of the parents showed c.640G < A (p.E214K) segregating from the father and c.248C < T (p.S83L) from the mother. In the medical literature, there are three pathogenic reports for the variant ‘c.640G > A,’ and for the variant ‘c.248C > T,’ there is one potential pathogenic case report.

Unfortunately, there are still no successful curative treatments for most cases of complex I deficiency. The primary approach for the majority of patients continues to be symptomatic measures. However, it has been known for almost two decades that some individuals, especially those with a myopathic presentation, might exhibit a clinical improvement through riboflavin (B2) supplementation [6]. Antioxidants such as vitamin E and coenzyme Q10 and their analogues are promising treatment strategies. Bezafibrate and AICAR, both of which activate PGC1α, were recently demonstrated to enhance multiple aspects of mitochondrial function in cultured skin fibroblasts derived from patients with nuclear-encoded complex I deficiency [6]. In our case, the patient was given riboflavin, biotin, thiamine, levodopa, levocarnitine, and coenzyme Q10.

We conducted a literature search of the PubMed database using filters for English-language case reports. The search used the keyword (NDUFV1). We excluded irrelevant articles and those that were inaccessible, and ultimately reviewed 11 articles. We summarized the characteristics of these articles in (Table 2).

**Table 2:** Literature review with similar cases of compound heterozygous NDUFV1 mutation.

Mutation and inheritance	Age of onset/Sex	Symptoms	Imaging	Treatment	Reference
Compound heterozygous mutation: Maternal c.1157G > A (p.Arg386His) and paternal c.1080G > A (p.Ser360=)	1 y/o, male	Developmental delay, staring and responsive episodes, EEG changes typical for absence seizures (1 y/o) Dysarthria and unsteady walking (2 y/o) Left exotropia associated with amblyopia, stroke-like episodes with acute onset of asymmetric weakness and coordination issues that persisted for a number of days before resolving (3 y/o) Bilateral optic atrophy (4 y/o) Severe headaches, tongue dysarthria, loss of communication skills and the ability to write, mild diplegic gait (5 y/o)	Brain MRI: Bilateral symmetrical increased T2 signal in the medial thalami with abnormal signal also seen in the midbrain and tectal plate. Later multiple new T2 hyperintense lesions without restricted diffusion involving the cervical cord, dorsal medulla and pons and central midbrain, new signal abnormalities in the substantia nigra	Ubidecarenone 150 mg twice daily, riboflavin 100 mg three times a day and thiamine 100 mg three times a day	Kiss et al. 2023 (36896486) [11].
Compound heterozygous mutation: c.365C > T (p.P122L) and c.565 T > C (p.S189P) inherited from each parent	24 y/o, female	Paresthesias, numbness, and progressive symmetric weakness involving all extremities, upper motor neuron type weakness in all extremities, positive Babinski sign, and decreased sensation to light touch in the left upper extremity, episodes of severe lactic acidosis, worsening encephalopathy, and vomiting	Brain MRI: Right frontal ring enhancing lesion with surrounding edema and long segment T2-weighted spinal cord signal abnormality extending from C1-T2 concerning for a demyelinating process. Progression of cystic encephalomalacia involving the bilateral frontal lobes (Fig. 1a), periventricular white matter (Fig. 1c), and corpus callosum, which largely spared the grey matter Spine MRI: Interval progression of T2/STIR signal along the central and dorsal thoracic cord extending to the conus medullaris	High dose intravenous (IV) steroids, plasmapheresis and rituximab. Bicarbonate infusion and eventually initiation of continuous renal replacement therapy for lactic acidosis.	Becker et al. 2022 (36163075) [2].
Compound heterozygous mutation: Maternal c.118C > T (p.Arg40Trp) and paternal c.1157G > A (p.Arg386His)	4 m/o, male	Diarrhea, delayed development, loss of head control, hypotonia, high fever, hoarseness, cardiorespiratory arrest, decreased heart rate, seizures, strabismus and weakened deep tendon reflexes	Brain MRI: Bilateral symmetric T2 hypersingal lesions in medulla oblongata, pons, midbrain and cervical spinal central with T1 hyposingal lesions in above areas	N/A	Tang et al. 2022 (35482246) [1].
Homozygous mutation: c. 365C > T (p.Pro122Leu), inheritance from both parents (consanguineous parents at the 3rd degree)	4 m/o, male	Delay of motor development, generalized hypotonia and brisk reflexes (4 m/o) Progressive spastic paraplegia and signs of cerebellar ataxia during his first two decades. Intellectual disability (IQ around 60). Fatigue, dysarthria, difficulties lifting his upper limbs, drowsiness, excessive sweating, severe dysarthria, generalized muscle weakness, and areflexia (40 y/o)	Brain MRI: in the T2-weighted image an inhomogeneous hypersignal in the posterior cervical region, extending up to the anterior horn. The brain was of normal size and not atrophic, however all ventricles were dilated symmetrically. The white matter showed diffuse T1-hypointensities and T2-hyperintensities, mostly in the frontal periventricular regions, sparing gray matter, and U fibers. Cysts with partially enhancing walls and hyperintense areas gave a heterogeneous appearance to the thinned diseased white matter. MR spectroscopy: highly elevated lactate peak within the frontal white matter lesions, as well as a moderately decreased peak of N-acetylaspartate (NAA)	Oral baclofen for spasticity. IV methylprednisone 1 g/d for 6 days for a suspected acute disseminated encephalomyelitis	Gschwind et al. 2022 (35482023) [12].
Compound heterozygous mutation: Maternal c.640G > A (p.Glu214Lys) and paternal c.1207dupG (p.Asp403Glyfs*27)	3 y/o, male	Developmental delay, autistic spectrum disorder, episodic vomiting and dehydration associated with a cutaneous rash, respiratory insufficiency, absence seizures, apnea	Brain MRI: T2-weighted hyperintense and T1-weighted hypointense focal lesions in central portions of the upper segment of the cervical spinal cord, with additional symmetric lesions in midbrain, pons, and bulb. Increasing intensity of lesions in a follow-up MRI a month later MR spectroscopy: elevation of lactate	Coenzyme Q10 (CoQ) (300 mg/day) and L-carnitine (100 mg/kg/day)	Zanette et al. 2021 (34807224) [3].
Homozygous mutation: c.1268C > T (p.Thr423Met), inheritance from both parents (first-degree cousins)	4 m/o, male	Minor cervical hypotonia and mild right hemibody hypertonia (4 m/o). Developmental delay, aggressive behavior, learning difficulties, motor regression, lethargy, irritability and apnea (7 y/o) Optic atrophy, sensorineural deafness, ptosis, hypotonia, hyperreflexia with diplegic spasticity, dysphagia and hyperhidrosis (11 y/o)	Brain MRI: Hyperintensity in T2/FLAIR/T2 in the thalamus, lentiform nucleus, frontal lobe. MR spectroscopy: Lactate peak	CoQ (300 mg/ day), L-carnitine (2 g/day), carbamazepine (17.5 mg/kg/day), biotin (20 mg/day), creatine (2 g/day), clobazam (0.4 mg/kg/ day), baclofen (15 mg/day), vitamin C and B complex	
Compound heterozygous mutation: Maternal c.1268C > T (p.Thr423Met) and paternal c.766C > T (p.Arg256Cys)	9 m/o, female	Progressive hypotonia and somnolence, gradual loss of motor skill, lethargy and dysphagia (9 m/o) Loss of neurodevelopmental milestones, hypotonia, strabismus and severe inappetence (3 y/o)	Brain MRI: Diffuse white matter lesions MR spectroscopy: lactate peak	CoQ, creatine, sodium bicarbonate, acetyl L-carnitine, riboflavin, and biotin	
Compound heterozygous mutation: Maternal c.756delC (p.Thr253Glnfs*44) and paternal c.1156C > T (p.Arg386Cys)	7 m/o, female	Hypotonia, spasticity, myoclonus, nystagmus, and reduced deep tendon reflexes (7 m/o) Slurring of speech, hyperacusis, hepatomegaly, and gradual loss of subcutaneous fat (9 m/o)	Brain MRI: Periventricular white matter lesions, irregular signal abnormalities in the putamen, caudate nucleus, corpus callosum, anterior and posterior limb of the internal capsule, thalamus, pontine and medullary pyramidal tract and medial lemniscus. Various other signal abnormalities.	N/A	Borna et al. 2020 (33182419) [13].
Homozygous mutation: c.1268C > T (p.Thr423Met), inheritance from both parents (first-degree relatives)	9 y/o, male	Gait ataxia, horizontal nystagmus, dysarthria, bilateral dysmetria, intention tremor, and dysdiadochokinesia	Brain MRI: Bilateral, symmetric, hyperintense signal in the putamen and right caudate nucleus on T2-weighted imaging MR spectroscopy: high lactate peak in the affected areas	N/A	Incecik et al. 2018 (30090137) [14].
Homozygous mutation: c.1118 T > C (p.Phe373Ser), inheritance from both parents (third-degree consanguineous parents)	6 m/o, male	Developmental delay, excessive cry, seizures, momentary loss of head control, myopia, bilateral lower set ears, nystagmus, mosaic pigmentary anomalies, hepatomegaly, and spasticity in lower limbs, extreme plantar responses and brisk deep tendon reflexes.	Brain MRI: diffuse hyperintensity in the cerebral white matter, cerebellar white matter and brainstem white matter, and small cystic areas in the periventricular white matter MR spectroscopy: reduced N-acetyl aspartate and elevated choline levels and an inverted double peak of lactate	N/A	Srivastava et al. 2018 (29976978) [5].
Homozygous mutation: c.1156C > T (p.Arg386Cys), inheritance from both parents (third-degree consanguineous parents)	6 y/o, male	Neuroregression, mild cognitive decline with regressive speech deficiencies, bilateral optic atrophy, and marked motor decline. History of seizures. Spasticity in all four limbs, clonus, and nystagmus.	Brain MRI: diffuse white matter demyelination with cystic areas consistent with neurodegeneration	N/A	
Compound heterozygous mutation: c.1156C > T (p.Arg386Cys) and 5′ splice site variation in intron 2 c.155 + 1G > G/A	1 y/o, male	Loss of achieved motor milestones, wasting, severe stunning, spasticity of all four limbs, brisk deep tendon reflexes and extensor plantar response, bilateral horizontal nystagmus.	Brain MRI: diffuse cystic leukoencephalopathy involving corpus callosum, deep and periventricular white matter with sparing of basal ganglia, brainstem, and cerebellum. MR spectroscopy: lactate doublet at 1.3 ppm	Riboflavin, coenzyme Q, vitamin E, thiamine, leucovorin, and carnitine	Wadhwa et al. 2018 (29948731) [15].
Heterozygous mutation: Maternal c.1268C > T, p.(T423M). Simultaneous homoplasmic ND1 variant m.3460G > A, another complex I subunit causing Leber Hereditary Optic Neuropathy (LHON)	Not specified, female	Decreased exercise tolerance and muscle weakness (early childhood). Signs of depression, physical fatigue, insomnia, aphonia and sudden change in personality (11 y/o) Progressive myopathy with severe weakness, inability to stand up, and wheelchair dependence. Aggravation and earlier onset of LHON compared to her siblings	Brain and spinal cord MRI: no abnormalities	N/A	Baertling et al. 2018 (29395179) [7].
Compound heterozygous mutation: Maternal c.640G > A (p.Glu214Lys) and paternal c.1162 + 4A > C (p.Glu214)	2 y/o, female	Febrile convulsions, partial left ptosis. Progressive asymmetric bilateral dystonia of her hands and feet (7 y/o) Wheelchair dependence (10 y/o) Resting tremor, dysarthria and dysphagia (12 y/o)	Brain MRI: symmetrical putaminal lesions, involvement of the left body of the caudate and the right quadrigeminal plate MR spectroscopy: lactate peak	Thiamine, coenzyme Q10, l-carnitine and biotin	Nafisinia et al. 2017 (27344648) [16].

## Conclusion

Our research unveils a hitherto undiscovered pathogenic effect of the variant ‘c.248C > T’ in the NDUFV1 gene, marking a significant contribution to the medical literature. While this study provides a pivotal starting point, the journey toward effective treatment necessitates further investigations into the broader landscape of NDUFV1 gene variants. The ultimate goal is to translate this newfound knowledge into targeted therapeutic strategies, thereby offering hope for individuals affected by diseases associated with the NDUFV1 gene.

## Consent to participate

Written informed consent was obtained from the patient’s parents for the anonymized information to be published in this article.

## Consent for publication

Written informed consent was obtained from the patient’s parents for the anonymized information to be published in this article.

## Supplementary Material

WES_child_omae166

WES_parents_omae166

## Data Availability

Not applicable.
