# Supplementary material for: Mitochondrial complex I deficiency in a 4-year-old boy due to compound heterozygous NDUFV1 mutation: a case report of a new pathogenic variant
Source: Oxf Med Case Reports. 2025 Apr 8;2025(4):omae166. doi: 10.1093/omcr/omae166 (PMC11979451; doi:10.1093/omcr/omae166)

GENIAN

Genetik Hastaliklar Degerlendirme Merkezl
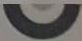


Moleküler Genetik Analiz Raporu

Adi Soyadi :HASAN SEYHO Numune Türu :Periferik Kan

Doğum Tarihi :15.08.2019 Istek Tarihi/Saati :16.06.2023/14:22:58

Cinsiyet : Erkek Örnek Alim Tarihi/Saati :14.06.2023/14:23:28

T.C.No :34*******38 Örnek Alim Yeri :Kurumdan Gönderildi

Protokol No /Lab No: 188539/ MG16227/23 Lab.Teslim Tarihi/Saati :16.06.2023/14:23:17

Gönderen Hekim :- Rapor Tarihi/Saati :08.09.2023/13:59:08

Gönderen Kurum :Diyarbakir Bireysel Rapor No :1188539.39141.2023/RO

Endikasyonu :Ensefalit?,Sol santral tip fascial paralizi, Sağ kol ve sağ bacakta güçsüzlük, Dengesini sağlayamakta

olgunun bulgulan lle ilskili OMIM ve HPO genleri incelenmistir. Ekzon-intron bağlanti noktalari (±10bc)analize dahil Elde edilen verilerin patojenisite siniflandirmasi "ACMG Guideline'a (PMID:25741868)ve ikincil bulgularin (insidental)analizi ACMG SFv3.1'e gore (PMID:35802134)yapilmiştir.

Sonuc: Başvuru endikasyonu ile iliskilendirilebilecek genetik değisimler aşağidaki Tabloda sunulmustur.

Asağidaki Tabloda belirtilen varyantlarin tamami icin Sanger dizileme ile doğrulama çalişmalarinin yapilmasi önerilir.

| Gen  Transkript | Pozisyon | Gen ile iliskili hastalik | Değisim  (Varyant) | Genotip | Yorum  (ACMG Sinifi) |
| --- | --- | --- | --- | --- | --- |
| NDUFV1  NM_007103.4 | chr11:67377981  (Exon 1) | Mitochondrial complex 1 deficiency,nuclear type 4 \|AR | Missense Variant,  Nmd Transcript  Variant  c.640G>A  (p.E214K)  rs121913661 | Heterozigot | Patojenik  (PP5-PP3-PM1-PM2) |
|  | chr11:67376115  (Exon 3) |  | Missense Variant  c.248C>T(p.S83L)  rs779150755 | Heterozigot | Olasi patojenik  (PP5-PP3-PM1-PM2) |

AD:Otozomal dominant,AR: Otozomal Resessif

Yorum:

NDUFVI geninde heterozigot, Patojenik/Olasi patojenik değisimler saptanmıştır.

Clin Var veri tabaninda "c.640G>A" değişimi için 3 adet patojenik bildirim,"c.248C>T" değisimi icin ise 1 adet olasi patojenik, 1 adet VUS bilirim yer almaktadir. Olgunun bulgularının otozomal resesif geçiş gösteren "Mitochondrial complex I deficiency, nuclear type 4 (OMIM #618225)"kliniği açısından değerlendirilmesi, aile taramasi ve olgunun rapor hakkinda genetik danismanlik almasi onerilir.

On tani da belirtilmis olan;RANBP2 geninin kodlayici bölgeleri icin 20X okuma derinliğinde hedef kaplam oranı %95'in üzerindedir ve bu gene ait patojenik değisim saptanmamistir. Tabloda yer alan varyantin neden olduğu hastalik (Mitochondrial complex I deficiency, nuclear type 4| AR)olgunun kliniğini açıklamadığı takdirde,olgu icin mtDNA tüm gen dizi analizi calismasi önerilir.

-Yukaridaki Tablolarda sunulan değişimlerin olgunun klinik bulguları ile birlikte değerlendirilmesi ve genetik danışma verilmesi önerilir.

-Genetik değisimlerin sunulduğu Tablolarda ACMG sinifi sütununda, tespit edilen değisimlerin hastaliklar ile iliskisi paylasilmaktadir.

Patojenik (P), Olasi Patojenik (LP) genetik değisimler (varyantlar) olgunun klinik yonetimini etkileyebilecek olanlardir.

Literatürde,hakkinda yeterli bilgi bulunmayan değisimler ise "VUS"(klinik önemi bilinmeyen varyant) olarak rapor edilmektedir.

Literatürün sürekli olarak yenilenmesi ve genislemesi nedeniyle,verilerin periyodik olarak analizleri taniya katki sağladiğindan (PMID:29095811); VUS değisimler icin 1 yil sonra analiz sonuclarinin tekrar degerlendirilmesi önerilir.

Daha once siniflandinilmis bir değisimin güncellenen bilgiler doğrultusunda yeniden değerlendirildiğinde baska bir sinifta yer alabileceği bilgisi klinik değerlendirmede dikkate alinmalidir.

Yapilan analiz,testin yapildigi bireyde hastaligin sebebini ortaya cikarmak amaciyla yapilmaktadir; otozomal resesif hastaliklarda tasiyialik durumu (heterozigotluk) ozel durumlar haricarastinlmamaktadir.

- Calisma, MGI DNBSEQ-G400 platformu kullanilarak yapilmis ve Genomize Seq,Software Version 6.6.0. programi ile analiz edilmistir.Testin dogruluk orani yontemin kapsama oranina ve Çalisilan DNA bölgesinin özelliğine gore değişiklik gosterebilir.

Bu çalışma ile büyük delesyon ve duplikasyonlar, intronik bölge ve UTR (untranslated region)mutasyonlan, uniparental dizomi ile meydana gelen hastaliklar, gonadal mozaisizm,sitogenetik anomaliler tespit edilmemektedir.

Analiz sonucunda tespit edilip raporlanan degisimler "dogum oncesi tabanlarindaki mevcut güncel bilgiler dikkate alinarak yapilmaktadir.Veri tabanlanindaki bilgiler zaman icerisinde güncellenmektedir


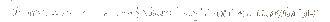

Supplement: WES_child_omae166 [file wes_child_omae166.docx]
