# Supplementary material for: Mitochondrial complex I deficiency in a 4-year-old boy due to compound heterozygous NDUFV1 mutation: a case report of a new pathogenic variant
Source: Oxf Med Case Reports. 2025 Apr 8;2025(4):omae166. doi: 10.1093/omcr/omae166 (PMC11979451; doi:10.1093/omcr/omae166)
Supplement: WES_parents_omae166 [file wes_parents_omae166.pdf]

GENTAN

Genetik Hastalıklar Değerlendirme Merkezi

Moleküler Genetik Analiz Raporu

**Adı Soyadı**	: SEVSEN ŞEYHO	**Numune Türü**	: Periferik Kan
**Doğum Tarihi**	: 05.01.1991	**İstek Tarihi / Saati**	: 28.09.2023 /17:13:49
**Cinsiyet**	: Kadın	**Örnek Alım Tarihi / Saati**	: 28.09.2023 /17:14:04
**T.C. No**	: 25*******04	**Örnek Alım Yeri**	: Kurumdan Gönderildi
**Protokol No / Lab No**	: 203407 / MG26815/23	**Lab. Teslim Tarihi/Saati**	: 28.09.2023 /17:13:55
**Gönderen Hekim**	: Uzm. Dr. Murat Öztürk	**Rapor Tarihi/Saati**	: 31.10.2023 /11:17:40
**Gönderen Kurum**	: Batman Eğitim ve Araştırma Hastanesi	**Rapor No**	: 1203407.39111.2023 / R0
**Endikasyonu**	: Genetik Taşıyıcılık Şüpheli Birey Taraması (Q14.8)		
**Test Adı**	: **Bilinen Mutasyon Analizi 1**		

İşlem: Hasta materyalinden elde edilen DNA örneği, çalışılması için istemi yapılan hedef bölgeye yönelik dizayn edilmiş spesifik primerler ile kullanılarak dizi analizi yöntemi ile çalışılmış ve analiz edilmiştir. Elde edilen verilerin patojenisite sınıflaması *ACMG Guideline*'a (PMID: 25741868) göre yapılmıştır. Referans genom hg19'dur.

Sonuç:

Saptanan varyantlar:

NDUFV1 (NM_007103.4) geninde HETEROZİGOT c.248C>T (p.S83L) rs779150755 değişimi **saptanmıştır.**

Saptanmayan varyantlar:

NDUFV1 (NM_007103.4) geninde c.640G>A (p.E214K) rs121913661 değişimi saptanmamıştır.

NOT: Sonucun genetik danışmanlık eşliğinde alınması önerilir.

Biyolog Ekrem [illegible]SIZOĞLU
Moleküler Genetik
Laboratuvar Sorumlusu

Prof. Dr. Ferda Emriye PERÇİN
Tıbbi Genetik Uzmanı
Dip. Tes. No: 4843

Bu rapor, laboratuvarın yazılı izni olmadan kopyalanıp çoğaltılamaz. İmzasız raporlar geçersizdir
Bu sonuçlar sadece incelemesi yapılan numune ile ilgilidir. Bu rapor dijital olarak imzalanmıştır.
^TÜRKAK tarafından TS EN ISO 15189 Standardına göre akredite edilmiştir.

GenQA INSTAND CAP

Kazım Dirik Mah. Üniversite Cad. No: 79/11 Bornova/İZMİR Tel: 0232 463 82 87 -0232 465 00 19 Fax: 0232 463 64 82 www.gentan.com
Ruhsat No:GHDM-SM/35.01/01

CamScanner ile tarandı

Moleküler Genetik Analiz Raporu

**Adı Soyadı**	: İDRİS ŞEYHO	**Numune Türü**	: Periferik Kan
**Doğum Tarihi**	: 03.05.1987	**İstek Tarihi / Saati**	: 28.09.2023 /17:15:58
**Cinsiyet**	: Erkek	**Örnek Alım Tarihi / Saati**	: 28.09.2023 /17:16:12
**T.C. No**	: 25*******40	**Örnek Alım Yeri**	: Kurumdan Gönderildi
**Protokol No / Lab No**	: 203409 / MG26816/23	**Lab. Teslim Tarihi/Saati**	: 28.09.2023 /17:16:04
**Gönderen Hekim**	: Uzm. Dr. Murat Öztürk	**Rapor Tarihi/Saati**	: 31.10.2023 /11:19:13
**Gönderen Kurum**	: Batman Eğitim ve Araştırma Hastanesi	**Rapor No**	: 1203409.39111.2023 / R0
**Endikasyonu**	: Genetik Taşıyıcılık Şüpheli Birey Taraması (Q14.8)		
**Test Adı**	: **Bilinen Mutasyon Analizi 1**		

İşlem: Hasta materyalinden elde edilen DNA örneği, çalışılması için istemi yapılan hedef bölgeye yönelik dizayn edilmiş spesifik primerler ile kullanılarak dizi analizi yöntemi ile çalışılmış ve analiz edilmiştir. Elde edilen verilerin patojenisite sınıflaması *ACMG Guideline*'a (PMID: 25741868) göre yapılmıştır. Referans genom hg19'dur.

Sonuç:

Saptanan varyantlar:

NDUFV1 (NM_007103.4) geninde HETEROZİGOT c.640G>A (p.E214K) rs121913661 değişimi **saptanmıştır.**

Saptanmayan varyantlar:

NDUFV1 (NM_007103.4) geninde c.248C>T (p.S83L) rs779150755 değişimi saptanmamıştır.

NOT: Sonucun genetik danışmanlık eşliğinde alınması önerilir.

Biyolog Ekrem [illegible]SIZOĞLU
Moleküler Genetik
Laboratuvar Sorumlusu

Prof. Dr. Ferda Emriye PERÇİN
Tıbbi Genetik Uzmanı
Dip. Tes. No: 4843

Bu rapor, laboratuvarın yazılı izni olmadan kopyalanıp çoğaltılamaz. İmzasız raporlar geçersizdir
Bu sonuçlar sadece incelemesi yapılan numune ile ilgilidir. Bu rapor dijital olarak imzalanmıştır.
^TÜRKAK tarafından TS EN ISO 15189 Standardına göre akredite edilmiştir.

Kazım Dirik Mah. Üniversite Cad. No: 79/11 Bornova/İZMİR Tel: 0232 463 82 87 -0232 465 00 19 Fax: 0232 463 64 82 www.gentan.com
Ruhsat No:GHDM-SM/35.01/01

CamScanner ile tarandı
